# Non-Parametric Kinematic Optimization of Flapping Foil Propulsion Using a Discrete Adjoint Method

**DOI:** 10.3390/biomimetics11060393

**Published:** 2026-06-03

**Authors:** Zhaoran Yin, Chao Zhou, Xiaofei Wang, Xiaocun Liao, Jian Wang

**Affiliations:** 1 The Laboratory of Cognition and Decision Intelligence for Complex Systems, Institute of Automation, Chinese Academy of Sciences, Beijing 100190, China; yinzhaoran2021@ia.ac.cn (Z.Y.); jianwang@ia.ac.cn (J.W.); 2 School of Artificial Intelligence, University of Chinese Academy of Sciences, Beijing 100049, China; 3 The Department of Automation, Tsinghua University, Beijing 100084, China; xiaofei-wang@mail.tsinghua.edu.cn

**Keywords:** flapping-foil, robot fish, discrete adjoint, Morison equation

## Abstract

Optimizing flapping-foil kinematics for underwater propulsion is challenging due to strong temporal coupling and nonlinear fluid–structure interactions. Most existing approaches rely on parameterized motion profiles, which restrict the accessible design space. A non-parametric kinematic optimization framework based on the discrete adjoint method is developed, enabling direct optimization of time-resolved motions without predefined functional forms. A Morison-based low-order hydrodynamic model, calibrated against Computational Fluid Dynamics (CFD), is employed for efficient evaluation within a validated regime. Results show that optimized motions substantially enhance propulsion performance over conventional sinusoidal motions, yielding non-sinusoidal, high-efficiency kinematics. In thrust-maximization cases, the optimized kinematics achieve a 50.29% increase in mean thrust by redistributing heave and pitch amplitudes and timing. Under balanced thrust–power conditions, the optimized motions consistently outperform sinusoidal counterparts. In power-minimization cases, a “generator-like” regime emerges, indicating a reversal of net energy transfer enabled by the non-parametric formulation. These results demonstrate that non-parametric optimization provides enhanced design flexibility and improved propulsion performance, offering a practical framework for biomimetic underwater propulsion design.

## 1. Introduction

Bio-inspired flapping propulsion has garnered significant and sustained interest in fluid mechanics and biomimetic robotics, and has been increasingly explored for underwater propulsion applications. This attention stems from its exceptional maneuverability and high propulsive efficiency under low Reynolds number and strongly unsteady flow conditions [1,2,3]. In contrast to conventional propeller-based systems, flapping foils generate thrust through periodic pitching and heaving motions, giving rise to pronounced unsteady inertial and drag effects in the surrounding fluid [4,5]. The performance of such propulsion mechanisms, however, is highly sensitive to the temporal characteristics of the prescribed kinematics. Variations in phase relationships, acceleration profiles, and the timing of velocity peaks can substantially influence both thrust production and energetic efficiency [6,7]. Consequently, within practical constraints, the systematic identification of high-performance unsteady motion patterns remains a challenging and open problem.

In existing studies, the Morison equation has been widely adopted as a reduced-order approximation for modeling hydrodynamic forces on slender structures or thin foils undergoing unsteady motions, owing to its clear physical formulation and high computational efficiency. It has found extensive application in areas such as wave-induced load estimation and the hydrodynamic modeling of biomimetic underwater robots [8,9,10,11]. Compared with high-fidelity Computational Fluid Dynamics (CFD) approaches, low-order models based on the Morison equation can capture the dominant added-mass and drag contributions to unsteady hydrodynamic forces at a substantially lower computational cost, rendering them particularly suitable for parametric studies, control analysis, and optimization problems [12]. However, due to its reduced-order nature, the Morison formulation does not explicitly resolve detailed vortex dynamics, such as vortex shedding, wake interactions, and flow-history effects associated with strongly separated unsteady flows.

The applicability of the Morison model in unsteady propulsion problems is inherently limited by the variability of its empirical parameters. Numerous studies have shown that these parameters are not universally valid, but instead remain reliable only within a restricted range of motion amplitudes, frequencies, and phase combinations, typically determined through experimental measurements or high-fidelity numerical simulations [13,14,15]. When kinematic patterns deviate significantly, particularly in terms of phase relationships or acceleration timing, the original parameter set often loses predictive accuracy, resulting in a marked decline in model performance. This limitation poses a fundamental challenge for applications involving kinematic variation or optimization, where small changes in phase and timing can significantly degrade model accuracy and control performance. Similar challenges of phase-dependent uncertainty and sampling sensitivity have been widely reported in precision motion measurement and control systems, where adaptive sampling and phase-adjustment strategies are often required to mitigate temporal interference and improve robustness [16,17]. Therefore, rather than seeking a universal parameter set, the present work adopts a localized parameter-identification strategy based on high-fidelity CFD simulations, with the objective of improving the predictive capability of the reduced-order model within the motion space relevant to the optimization problem. The optimized kinematics are subsequently re-evaluated in the CFD environment to further assess the resulting propulsion performance under more realistic unsteady flow physics.

For the performance optimization of flapping propulsion, existing studies predominantly parameterize kinematic inputs into a finite set of variables. For instance, Gehrke et al. [18] represented half-stroke pitching motion with four control points and applied a genetic algorithm to separately maximize cycle-averaged efficiency and lift. Liu et al. [19] employed a multifidelity evolutionary algorithm to optimize five kinematic parameters of a flapping energy harvester in both single-objective (efficiency) and bi-objective (efficiency–power) scenarios. Wang et al. [20] introduced a Bayesian-optimization-based multitask, multifidelity Gaussian process framework for the parameterized prediction and optimization of thrust and propulsive efficiency.

These parameterized strategies offer engineering appeal due to their intuitive formulation and straightforward implementation. However, they rely on a predefined functional representation of the kinematics, which inherently restricts the admissible motion space and may limit the ability to represent complex optimal trajectories unless the number of parameters is significantly increased, leading to a rapidly growing optimization dimensionality. As a result, their fundamental limitation lies in the inherent prior assumption imposed on the solution space by the chosen parameterization. If the true optimal motion falls outside the prescribed function family, even the best solution within that parametric space may substantially deviate from the global optimum. This issue is especially critical in engineering contexts with input power or actuator constraints. To pursue higher mean thrust, parameterized models often aggressively increase motion amplitude or frequency [21,22], potentially leading to instantaneous power demands beyond physical limits. Consequently, systematically identifying high-performance unsteady motions, without predefined kinematic forms and within feasible power bounds, remains an ongoing research effort.

Several studies have explored non-parameterized optimization methods to overcome the expressiveness limitations of parameterized descriptions. Here, “non-parameterized” refers to approaches in which the control or design variables are directly treated as time-resolved quantities, rather than being restricted to a prescribed functional form. Nieto et al. [23] proposed a convex-optimization-based non-parameter design framework for addressing energy consumption and peak-power issues in periodic motions driven by series elastic actuators. In their approach, the force–displacement relationship of the elastic element was directly treated as the optimization variable, enabling energy-efficiency and power constraints to be considered without assuming a predefined functional form. For flapping propulsion, Wang et al. [24] employed deep reinforcement learning to conduct non-parameterized planning of flapping motions, using instantaneous foil kinematics as direct control inputs to autonomously learn periodic flapping trajectories. Their results showed that this method can produce non-harmonic flapping motions and improve propulsive performance without prescribing specific kinematic forms. However, such methods generally rely on extensive interaction samples and reward-driven exploration, which may become computationally inefficient in high-dimensional time-resolved control spaces. In addition, explicit physical constraints, such as input power or actuator limitations, are not directly embedded into the optimization process, making it more difficult to systematically enforce physically meaningful motion constraints during optimization.

The optimization of unsteady flapping kinematics is characterized by strong temporal coupling, as both objectives and constraints depend on period-integrated quantities over the entire motion cycle. This leads to a high-dimensional, time-discretized control problem, for which discrete adjoint methods provide an efficient and scalable gradient-based approach, enabling direct computation of sensitivities with respect to each discrete time-step control variable. Unlike parameterized approaches that optimize within a restricted basis, this formulation operates directly in the full time domain, making it well-suited for non-parametric trajectory optimization. Despite these advantages, the application of discrete adjoint methods to flapping kinematics optimization remains relatively limited, with most existing studies focusing either on aerodynamic shape optimization or simplified kinematic configurations. Abergo et al. [25] proposed an aerodynamic shape optimization framework that integrates a discrete adjoint method with a data-reduction-based radial basis function mesh deformation strategy. Under constraints such as lift preservation, volume conservation, and minimum thickness requirements, their approach demonstrated improved robustness over the linear elasticity analogy mesh deformation method and achieved a significant drag reduction without compromising lift. Xu et al. [26] applied an adjoint-based optimization approach to a hinged flapping-wing model equipped with a trailing-edge flap, optimizing the flap kinematic parameters to enhance lift performance in hovering conditions. Rutkowski et al. [27] developed a two-dimensional open-loop optimal control framework for flapping-wing motions based on an adjoint lattice Boltzmann method. By coupling an immersed boundary method to handle wing–flow interactions with B-spline parameterization of the flapping kinematics, and by considering mean lift and mechanical power as objective functions, they obtained a Pareto-optimal front, thereby demonstrating the effectiveness of adjoint-based approaches in complex flapping motion optimization problems. These studies demonstrate the effectiveness of adjoint-based optimization for unsteady flapping propulsion problems. However, applications to non-parameterized time-resolved kinematic optimization remain relatively limited due to the high dimensionality and computational complexity associated with long-horizon unsteady motion optimization.

Based on the above considerations, this study proposes a non-parameterized flapping kinematics optimization framework that integrates a Morison-type hydrodynamic model with a discrete adjoint method. Departing from most existing approaches, the method does not prescribe any functional form for the kinematics. Instead, pitching and heaving accelerations at discrete time steps are directly treated as control variables. The motion sequence over a half flapping period is optimized under periodicity, half-cycle antisymmetry, and input-power constraints. This non-parameterized formulation allows the search to move beyond conventional sinusoidal profiles and explore a broader space of unsteady kinematic solutions. In contrast to conventional low-dimensional parameterized optimization approaches, the proposed framework enables high-dimensional direct optimization of the temporal motion history, allowing strongly non-sinusoidal propulsion patterns to emerge naturally from the optimization process. To compensate for the limitations of low-order models under complex flow conditions, high-fidelity CFD simulations are employed as a high-fidelity reference model to perform targeted identification of the Morison model parameters. The optimized kinematics are subsequently evaluated in the CFD environment, enabling performance validation under more realistic unsteady flow physics.

Overall, the main contributions of this work can be summarized as follows:

1. A discrete-adjoint-based, non-parameterized flapping kinematics optimization framework is developed using the Morison equation, which enables efficient gradient computation for high-dimensional periodic flapping optimization problems without relying on predefined kinematic functions.
2. Input-power constraints are explicitly embedded in the optimization process, facilitating a balanced trade-off between propulsive performance and the practical realizability of the optimized propulsion kinematics under physically motivated power-related limitations.
3. The optimized results are systematically validated with high-fidelity CFD simulations, demonstrating the physical consistency and predictive capability of the proposed low-order-model-adjoint approach for unsteady flapping propulsion.

The remainder of this paper is structured as follows. Section 2 describes the kinematic modeling of the flapping system, the Morison hydrodynamic formulation, and the CFD-based parameter identification procedure. Section 3 presents the discrete adjoint method and the construction of the optimization problem. Section 4 discusses the optimization outcomes alongside CFD validation. Finally, Section 5 concludes the paper and outlines directions for future research.

## 2. Kinematics and Hydrodynamics

This section first presents the forward kinematic model and its discrete state update formulation, followed by the Morison-based hydrodynamic modeling and the computation of forces and input power. The high-fidelity CFD setup and the CFD-based parameter identification procedure are then introduced, providing a reliable physical basis and reference for the subsequent discrete adjoint optimization.

### 2.1. Forward Kinematics and State Update

**Figure 1:**
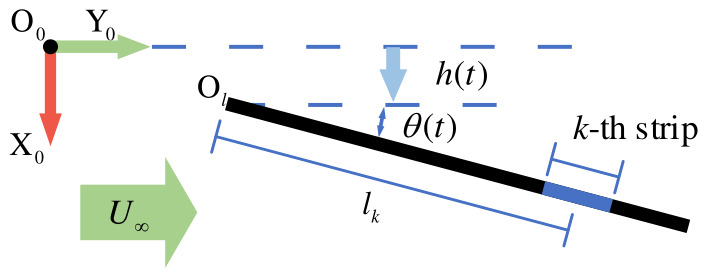
Definition of the imposed heave–pitch kinematics for the flapping foil.

For a bio-inspired robotic fish moving in the horizontal plane, the multi-link body can be equivalently represented as a set of foils flapping about axes normal to the plane of motion. Figure 1 illustrates a single foil, where the leading-edge point $O_{l}$ is chosen as the reference. The pitching angle θ(t) and the heaving displacement h(t) are defined accordingly. The system is subjected to a uniform incoming flow with constant velocity $U_{\infty }$ along the global $Y_{0}$-direction, forming a typical two-dimensional flapping propulsion configuration.

To facilitate the construction of the discrete adjoint equations, all kinematic variables are expressed in a discretized form. Within the Morison-equation-based hydrodynamic modeling framework adopted in this study, acceleration is a fundamental physical quantity that directly contributes to the fluid forces. Therefore, to ensure continuous differentiability of the system states over the entire flapping period and to allow smooth influence of the control inputs on the system dynamics, the control input at discrete time instant *j* is defined as $u_{j}={\left[{\ddot{\theta }}_{j},{\ddot{h}}_{j}\right]}^{T}$, where ${\ddot{\theta }}_{j}$ and ${\ddot{h}}_{j}$ denote the pitching and heaving accelerations, respectively.

To establish a state transition that is naturally consistent with the acceleration-based inputs, the system state vector is defined as $x_{j}={\left[{\dot{\theta }}_{j},\theta_{j},{\dot{h}}_{j},h_{j}\right]}^{T}$.

Given a time step size Δt, the kinematic equations are discretized using a semi-implicit Euler scheme, yielding the following state update relations:

(1) $$\left[\begin{array}{c} {\dot{\theta }}_{j+1}\\ \theta_{j+1}\\ {\dot{h}}_{j+1}\\ h_{j+1} \end{array}\right]=\left[\begin{array}{c} {\dot{\theta }}_{j}+\Delta t\;{\ddot{\theta }}_{j}\\ \theta_{j}+\Delta t\;{\dot{\theta }}_{j+1}\\ {\dot{h}}_{j}+\Delta t\;{\ddot{h}}_{j}\\ h_{j}+\Delta t\;{\dot{h}}_{j+1} \end{array}\right].$$

The above update can be written compactly as

(2) $$x_{j+1}=F_{j}\left(x_{j},u_{j},\Delta t\right),$$

where $F_{j}$ denotes the discrete state update operator mapping the current state and control input to the state at the next time step.

A complete flapping period is discretized using 2N time steps, such that the flapping period is given by T=2NΔt. In the subsequent optimization process, Δt is also treated as a design variable, enabling adjustment of the flapping period (i.e., the flapping frequency).

It is noted that the flapping motion considered in this study is reciprocating, and the net lateral force along the global $X_{0}$-direction over one period is required to vanish. Consequently, the kinematics must satisfy both periodicity and half-cycle antisymmetry conditions:

(3) $$x_{j+2N}=x_{j},$$

(4) $$x_{j+N}=-x_{j}.$$

These properties imply that the motion over a full period is completely determined by the first half-cycle. Therefore, in both the forward simulation and the discrete adjoint optimization, only the states and hydrodynamic responses over j=1,…,N need to be computed.

Under these constraints, the initial state $x_{1}$ of the full flapping cycle is uniquely determined by the control input sequence $u_{j}$, j=1,…,N, and the time step size Δt through the discrete update relations (Equation (1)). Specifically, the initial conditions are given by

(5) $$\begin{array}{cc} {\dot{\theta }}_{1} & =-\frac{\Delta t}{2}\sum_{j=1}^{N}{\ddot{\theta }}_{j},\\ \theta_{1} & =\frac{\Delta t^{2}}{4}\sum_{j=1}^{N}\left(2j-N-2\right)\;{\ddot{\theta }}_{j},\\ {\dot{h}}_{1} & =-\frac{\Delta t}{2}\sum_{j=1}^{N}{\ddot{h}}_{j},\\ h_{1} & =\frac{\Delta t^{2}}{4}\sum_{j=1}^{N}\left(2j-N-2\right)\;{\ddot{h}}_{j}. \end{array}$$

### 2.2. Morison Equation Formulation

In the present work, the Morison equation is employed as a reduced-order hydrodynamic model to approximate the dominant inertial and drag-related force contributions acting on the flapping foil. The formulation is intended to provide a computationally efficient surrogate for time-resolved kinematic optimization, rather than a high-fidelity description of detailed vortex dynamics or wake evolution.

For a flapping foil with chord length *l*, the foil is uniformly discretized along the chordwise direction into *n* strips, and the Morison hydrodynamic force acting on each strip is computed independently. As illustrated in Figure 1, in the body-fixed coordinate system, the chordwise position of the center of the *k*-th strip relative to the leading-edge point $O_{l}$ is given by

(6) $$l_{k}=\frac{l(2k-1)}{2n},\;k=1,\ldots ,n.$$

For the *k*-th strip, the normal velocity $v_{n_{k}}$ (perpendicular to the foil) and the tangential velocity $v_{t_{k}}$ (along the foil surface) are expressed as

(7) $$v_{n_{k}}=\dot{h}\cos\theta +U_{\infty }\sin\theta +\dot{\theta }\;l_{k},$$

(8) $$v_{t_{k}}=-\dot{h}\sin\theta +U_{\infty }\cos\theta .$$

Based on the Morison equation, the hydrodynamic forces generated by the *k*-th strip in the normal and tangential directions are given by

(9) $$F_{n_{k}}=-C_{a_{n}}\;\rho \;V_{n_{k}}\;{\dot{v}}_{n_{k}}-\frac{1}{2}\rho C_{D_{n}}S_{n_{k}}\left|v_{n_{k}}\right|v_{n_{k}},$$

(10) $$F_{t_{k}}=-C_{a_{t}}\;\rho \;V_{t_{k}}\;{\dot{v}}_{t_{k}}-\frac{1}{2}\rho C_{D_{t}}S_{t_{k}}\left|v_{t_{k}}\right|v_{t_{k}},$$

where $C_{a_{n}}$ and $C_{a_{t}}$ denote the added-mass coefficients in the normal and tangential directions, respectively, $C_{D_{n}}$ and $C_{D_{t}}$ are the corresponding drag coefficients, $V_{n_{k}}$ and $V_{t_{k}}$ represent the effective fluid volume associated with the strip motion, $S_{n_{k}}$ and $S_{t_{k}}$ denote the projected area of the strip, and ρ is the fluid density.

Therefore, the total normal force, tangential force, and moment acting on the entire flapping foil about the leading-edge point $O_{l}$ are given by:

(11) $$F_{n}=\sum_{k=1}^{n}F_{n_{k}},\;F_{t}=\sum_{k=1}^{n}F_{t_{k}},\;M=\sum_{k=1}^{n}F_{n_{k}}\;l_{k}.$$

By projecting the hydrodynamic forces onto the negative $Y_{0}$-direction of the global coordinate system, the thrust generated by the flapping foil can be expressed as

(12) $$F_{\mathrm{thrust}}=F_{n}\sin\theta -F_{t}\cos\theta .$$

The instantaneous input power required to sustain the periodic flapping motion is given by

(13) $$P_{\mathrm{in}}=-\left(F_{n}\cos\theta +F_{t}\sin\theta \right)\dot{h}-M\dot{\theta }.$$

It is worth noting that the first term in Equation (13) can be interpreted not only as the product of the resultant force and the heaving velocity, but also from a geometric perspective. Specifically, one may introduce an imaginary upstream link of length $l_{\mathrm{imag}}\to \infty$ ahead of the leading edge, whose angular velocity ${\dot{\theta }}_{\mathrm{imag}}$ satisfies $\dot{h}=l_{\mathrm{imag}}{\dot{\theta }}_{\mathrm{imag}}$. Under this interpretation, $P_{\mathrm{in}}$ can be viewed as the equivalent rotational power of this imaginary link about its leading edge.

In the subsequent kinematic optimization, a constraint is imposed on the input power. On the one hand, this ensures that the optimized flapping kinematics remain feasible from an engineering standpoint; on the other hand, it effectively prevents the optimization from converging to non-physical extreme motion patterns, such as generating thrust through excessively large heaving amplitudes or unrealistically high flapping frequencies, thereby preserving the physical and engineering relevance of the optimized results.

### 2.3. Parameter Identification of Morison Coefficients Using CFD

To validate the accuracy of the discrete hydrodynamic model established in this work and to provide reliable physical references for the Morison model coefficients, high-fidelity computational fluid dynamics (CFD) simulations are employed as the reference model. The unsteady flow around the flapping foil and the resulting hydrodynamic forces during periodic flapping are numerically solved. Based on these simulations, the unknown parameters of the low-order hydrodynamic model are calibrated using a parameter identification approach, such that the model can statistically approximate the high-fidelity CFD results.

In the CFD simulations, the computational domain is discretized using unstructured meshes, and the incompressible Navier–Stokes equations are solved with the finite-volume method:

(14) $$\begin{array}{cc} \nabla \cdot v & =0, \end{array}$$

(15) $$\begin{array}{cc} \frac{\partial v}{\partial t}+v\cdot \nabla v & =-\frac{1}{\rho }\nabla p+\nu \nabla^{2}v, \end{array}$$

where v denotes the velocity field, *p* is the pressure, and ν is the kinematic viscosity. Turbulence effects are modeled using the realizable *k*-ε turbulence model, which is adopted due to its robustness and computational efficiency for repeated unsteady simulations involving oscillatory and separated flows. In the present study, the CFD simulations are primarily employed for hydrodynamic-force data generation in the parameter-identification process of the reduced-order Morison model, as well as for subsequent validation of the optimized kinematics. Therefore, the primary objective is to obtain stable and reliable prediction of cycle-scale hydrodynamic force and power trends, rather than detailed turbulence-resolved reconstruction of fine-scale vortex structures. Although more advanced turbulence models, such as the Shear Stress Transport (SST) *k*-ω model, Detached Eddy Simulation (DES), or Large Eddy Simulation (LES), may provide improved resolution of complex vortex dynamics, their substantially higher computational cost is less suitable for the large number of simulations required during the calibration and validation stages of the present study. The pressure–velocity coupling is handled via the Semi-Implicit Method for Pressure Linked Equations (SIMPLE) algorithm.

**Figure 2:**
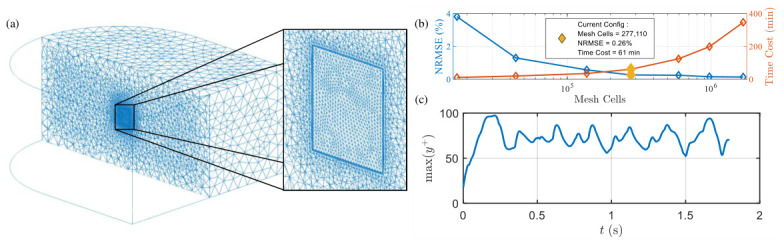
Computational mesh configuration. (**a**) Global view of the numerical grid with localized refinement around the moving plate. (**b**) Mesh resolution assessment. The influence of spatial discretization on the numerical results was examined by employing several computational grids with increasing levels of refinement. (**c**) Temporal evolution of the maximum dimensionless wall distance $y^{+}$ for the most demanding operating condition (f=2Hz, $h_{\mathrm{amp}}=0.5\;m$, and $\phi =90^{\circ }$).

To ensure computational accuracy while controlling the overall mesh size, mesh refinement is concentrated in the flapping region and its vicinity, with sufficiently dense nodes on the foil surface to accurately capture boundary layer characteristics and unsteady load variations. The computational fluid domain is defined as a rectangular region with dimensions of 2.0 m × 1.8 m × 0.6 m, as shown in Figure 2a. A uniform inflow velocity is prescribed at the inlet boundary, while a pressure outlet condition is applied at the downstream boundary to allow fully developed wake convection. The flapping foil is positioned sufficiently far from both inlet and outlet boundaries to minimize boundary-induced disturbances and ensure that wake development is not affected by domain truncation. Specifically, the background fluid domain employs a base mesh size of 0.4 m, while the dynamic mesh zone surrounding the flapping foil is refined to 0.06 m. The foil surface is further discretized using a fine mesh size of 0.002 m, which is smaller than the foil thickness, together with 7 inflation layers, to improve the near-wall flow resolution. The final computational mesh contains 55,806 nodes and 277,110 elements.

The heave-pitch motion of the flapping foil is applied to the rigid body boundaries within the computational domain via user-defined functions (UDFs), enabling precise control of the flapping process. It is important to emphasize that the CFD model does not directly participate in the subsequent kinematic optimization. Rather, it is employed solely to assess the accuracy of hydrodynamic predictions made by the low-order model under different kinematic conditions, and to validate the optimized kinematic schemes.

During the parameter identification stage, a nonlinear system identification approach based on a gray-box modeling framework is employed. The low-order hydrodynamic model, whose physical structure is known but parameters are unknown, is treated as a nonlinear dynamic system. Model parameters are determined by minimizing the discrepancy between the model predictions and the CFD-computed hydrodynamic responses.

The reference data for parameter identification are generated from a set of canonical sinusoidal flapping motions defined as

(16) $$\theta (t)=\theta_{\mathrm{kamp}}\;t\sin\left(2\pi ft\right),$$

(17) $$h(t)=h_{\mathrm{amp}}\;\sin\left(2\pi ft+\phi \right),$$

where $\theta_{\mathrm{kamp}}$ denotes the rate of increase in the pitching amplitude with time *t*, $h_{\mathrm{amp}}$ is the heaving displacement amplitude, *f* is the flapping frequency, and ϕ is the phase difference between the two motions. The use of $\theta_{\mathrm{kamp}}\;t$ to control the time-dependent growth of the pitching amplitude is intended to explore a wider range of motion states within a limited CFD simulation time.

The sensitivity of the numerical results to mesh resolution was examined through a mesh convergence study [see Figure 2b]. A representative operating condition was considered, with f=1Hz, $h_{\mathrm{amp}}=0.5\;m$, and $\phi =90^{\circ }$. The results indicate that the hydrodynamic force predictions are essentially independent of further mesh refinement. Compared with the finest grid, the selected mesh yields an NRMSE of 0.26%, while reducing the computational cost to about 61 min.

Since the present simulations adopt the enhanced wall treatment (EWT) in conjunction with the realizable *k*-ε turbulence model, the near-wall mesh resolution is evaluated using the dimensionless wall distance $y^{+}$, as shown in Figure 2c. A conservative assessment is performed under the most demanding operating condition considered in this study (f=2Hz, $h_{\mathrm{amp}}=0.5\;m$, and $\phi =90^{\circ }$), corresponding to the strongest unsteady case in the parameter space. The results indicate that the maximum $y^{+}$ remains below approximately 100 throughout the flapping cycle, while most values lie within the range of approximately 50–90 after the initial transient stage. These values are within the recommended applicability range of the EWT, confirming that the near-wall resolution is sufficient for the present simulations.

In addition, the sensitivity to temporal resolution was assessed. Since the present study considers flapping motions over a range of frequencies, the temporal resolution is characterized by the number of time steps per flapping cycle rather than by a fixed absolute time step size. Each flapping cycle is uniformly discretized into 100 time steps, following the temporal convergence analysis reported in our previous study [15], where this resolution was demonstrated to provide sufficient accuracy for capturing the unsteady hydrodynamic force evolution. Under the present frequency-varying framework, this strategy ensures a consistent temporal resolution across different operating conditions while maintaining computational efficiency.

Through the above CFD simulations and parameter identification procedure, a set of low-order hydrodynamic model parameters is obtained that captures the main trends of the high-fidelity numerical results, providing a reliable foundation for gradient evaluations of thrust and energy metrics in the subsequent discrete adjoint optimization.

## 3. Discrete Adjoint Method

In Section 2.1, the discrete-time control inputs and the corresponding state update operator of the flapping system have been defined. In Section 2.2, based on the Morison hydrodynamic model, expressions for the instantaneous thrust and input power generated by the flapping foil under prescribed kinematics have been derived. Building upon these formulations, this section presents the mathematical formulation of the flapping kinematic optimization problem, followed by the derivation of the corresponding discrete adjoint equations for efficient gradient evaluation.

### 3.1. Optimization Problem Formulation

The objective of this study is to investigate the trade-off between average propulsive performance and energy consumption under steady periodic flapping conditions. To this end, the following objective function is defined in the discrete-time framework:

(18) $$\min_{\left\{u_{j},\Delta t\right\}}J=\frac{2}{T}\sum_{j=1}^{N}L_{j}\left(x_{j},u_{j}\right)\;\Delta t,$$

where the stage cost at the *j*-th time step is defined as

(19) $$L_{j}=-\left(1-\beta \right)\;F_{\mathrm{thrust},j}+\beta \;P_{\mathrm{in},j}.$$

Here, β∈[0,1] is a weighting parameter that adjusts the relative importance between propulsive performance and energy consumption. The objective function represents a weighted combination of cycle-averaged thrust maximization and input power minimization. A smaller value of β biases the optimization toward enhanced thrust generation, whereas a larger value places greater emphasis on energy efficiency. By varying β, a family of Pareto-optimal solutions characterizing the trade-off between thrust and power can be systematically obtained.

Since a full flapping cycle consists of 2N discrete time steps and the cycle period satisfies T=2NΔt, the objective function can be further simplified as

(20) $$\min_{\left\{u_{j},\Delta t\right\}}J=\frac{1}{N}\sum_{j=1}^{N}L_{j}\left(x_{j},u_{j}\right).$$

This formulation indicates that, under the assumptions of periodicity and half-cycle antisymmetry, the optimization problem only requires summation over half of the flapping cycle. The objective function is therefore equivalent to the arithmetic mean of the stage costs over the half-cycle.

It is worth emphasizing that, although Δt does not appear explicitly in the objective, it governs the state evolution through the discrete operator $F_{j}$ and therefore indirectly affects the optimization by adjusting the flapping period.

To prevent the optimization from converging to non-physical motion patterns and to satisfy practical engineering constraints, penalty terms are introduced into the stage cost to account for excessive input power and heave displacement. The stage-wise penalty term is defined as

(21) $$L_{j}^{\prime }=\frac{1}{2}\;\sigma_{P_{\mathrm{in}}}\max\;{\left(0,\;P_{\mathrm{in},j}-P_{\max}\right)}^{2}+\frac{1}{2}\;\sigma_{h}\max\;{\left(0,\;|h_{j}|-h_{\max}\right)}^{2},$$

where $P_{\max}$ denotes the maximum allowable input power and $\sigma_{P_{\mathrm{in}}}$ is the corresponding penalty weight. Similarly, $h_{\max}$ represents the maximum admissible heave displacement, and $\sigma_{h}$ is the associated penalty coefficient.

The input power penalty term is inactive when $P_{\mathrm{in},j}\leq P_{\max}$, while it grows rapidly once the threshold is exceeded, thereby increasing the effective weight of the power term in the objective function and suppressing high-power solutions at the gradient level. The heave displacement penalty plays an analogous role in constraining excessive vertical excursions.

Accordingly, combining with the original stage cost defined in Equation (19), the total penalized stage cost is given by

(22) $$L_{t,j}=L_{j}+L_{j}^{\prime },$$

and the objective function with penalty terms becomes

(23) $$J_{t}=\frac{1}{N}\sum_{j=1}^{N}L_{t,j}.$$

Finally, to avoid abrupt changes in the pitch and heave accelerations, a smoothness regularization term is added,

(24) $$J_{s}=\frac{1}{2}\;\varepsilon_{\theta }\left[\sum_{j=1}^{N-1}{\left({\ddot{\theta }}_{j+1}-{\ddot{\theta }}_{j}\right)}^{2}+{\left({\ddot{\theta }}_{1}+{\ddot{\theta }}_{N}\right)}^{2}\right]+\frac{1}{2}\;\varepsilon_{h}\left[\sum_{j=1}^{N-1}{\left({\ddot{h}}_{j+1}-{\ddot{h}}_{j}\right)}^{2}+{\left({\ddot{h}}_{1}+{\ddot{h}}_{N}\right)}^{2}\right],$$

where $\varepsilon_{\theta }$, $\varepsilon_{h}$ are weighting coefficients. This term penalizes high-frequency variations in the prescribed kinematic profiles, thereby promoting smoother temporal evolution of the control inputs and suppressing excessive rapid acceleration variations during optimization. These constraints are introduced to improve the physical plausibility and practical realizability of the optimized kinematics while maintaining favorable propulsion performance. It should be noted, however, that detailed actuator-level constraints, such as motor torque limits and bandwidth restrictions, are not explicitly modeled in the present study.

For clarity, the complete optimization problem considered in this study can be summarized in the following standard form:

(25) $$\min_{\left\{u_{j},\Delta t\right\}}\;J_{f}=\frac{1}{N}\sum_{j=1}^{N}\left[L_{j}\left(x_{j},u_{j}\right)+L_{j}^{\prime }\right]+J_{s}$$

$$s.t.\;x_{j+1}=F_{j}\left(x_{j},u_{j},\Delta t\right),\;j=1,\ldots ,N.$$

Under the above objective function and dynamical constraints, the corresponding discrete adjoint equations and the gradient computation of the objective function with respect to the control inputs and the time step size will be derived in the following section.

### 3.2. Discrete Adjoint Derivation

To efficiently evaluate the gradient of the objective function with respect to the control variables, the discrete adjoint method is employed in this study. Unlike the continuous adjoint approach, the discrete adjoint method is constructed directly from the discrete-time state update equations, with each adjoint variable being associated with a corresponding discrete state.

To incorporate the discrete state update constraints, the discrete Lagrangian function is defined as

(26) $$L=\frac{1}{N}\sum_{j=1}^{N}L_{t,j}+\sum_{j=1}^{N}\lambda_{j+1}^{T}\left[F_{j}\left(x_{j},u_{j},\Delta t\right)-x_{j+1}\right],$$

where $\lambda_{j}\in R^{4}$ denotes the discrete adjoint variable associated with the state $x_{j}$.

Taking partial derivatives of the Lagrangian with respect to the state variables $x_{j}$, and enforcing the first-order optimality condition that the variation in the Lagrangian with respect to all free variables vanishes, yields the discrete adjoint recursion. For j=N,…,1, the adjoint variables satisfy the backward relation

(27) $$\lambda_{j}=\frac{1}{N}\frac{\partial L_{t,j}}{\partial x_{j}}+{\left(\frac{\partial F_{j}}{\partial x_{j}}\right)}^{T}\lambda_{j+1}.$$

Here, the Jacobian matrix $\partial F_{j}/\partial x_{j}$ characterizes the sensitivity of the state update operator to the current state, while $\partial L_{t,j}/\partial x_{j}$ reflects the dependence of the instantaneous thrust, input power, and penalty terms on the state variables.

Under the assumptions of periodic steady state and half-cycle antisymmetry, the objective function is defined over a half flapping cycle, and the terminal condition for the adjoint variables is given by

(28) $$\lambda_{N+1}=-\lambda_{1}.$$

Once the adjoint variables have been obtained, the gradient of the penalized objective function with respect to the discrete control inputs can be evaluated as

(29) $$\frac{\partial J_{t}}{\partial u_{j}}=\frac{1}{N}\frac{\partial L_{t,j}}{\partial u_{j}}+{\left(\frac{\partial F_{j}}{\partial u_{j}}\right)}^{T}\lambda_{j+1},\;j=1,\ldots ,N.$$

This expression shows that, at each discrete time step, the gradient consists of two contributions: one arising from the direct dependence of the stage cost on the control inputs, and the other accounting for the indirect influence of the controls on subsequent state evolution through the state update operator. This structure underlies the computational efficiency of the discrete adjoint method, as the gradient evaluation requires only one forward state integration and one backward adjoint integration, regardless of the dimension of the control space.

For the smoothness regularization term $J_{s}$, which acts solely on the control variables, no adjoint recursion is required. Its contribution to the gradient can be computed directly. Consequently, the gradient of the final objective function $J_{f}$ with respect to the discrete control inputs is given by

(30) $$\frac{\partial J_{f}}{\partial u_{j}}=\frac{\partial J_{t}}{\partial u_{j}}+\frac{\partial J_{s}}{\partial u_{j}}.$$

Within the discrete adjoint framework, the gradient of the final objective function with respect to the time step size can be written in a unified form as

(31) $$\frac{\partial J_{f}}{\partial \Delta t}=\frac{1}{N}\sum_{j=1}^{N}{\left(\frac{\partial F_{j}}{\partial \Delta t}\right)}^{T}\lambda_{j+1}.$$

Since the smoothness regularization term $J_{s}$ does not explicitly depend on Δt, the above expression involves only the penalized objective function through the state update operator. By treating Δt as an optimization variable while keeping the number of discrete time steps fixed, continuous adjustment of the flapping period is enabled, providing additional freedom for improving kinematic performance.

Based on the gradient information obtained from the adjoint method, the optimization problem is solved using a sequential quadratic programming (SQP) approach. At the *m*-th iteration, a quadratic programming subproblem is formulated using the adjoint-based gradient information, from which a search direction $d^{\left(m\right)}$ is computed. The design variables are then updated as

(32) $$z^{\left(m+1\right)}=z^{\left(m\right)}+\alpha^{\left(m\right)}d^{\left(m\right)},$$

where $z=\{u_{j},\Delta t\}$ denotes the set of all design variables and $\alpha^{\left(m\right)}$ is the step size determined by a line search procedure. The iteration is terminated when the change in the objective function or the norm of the adjoint-based gradient $\nabla J^{\left(m\right)}$ falls below a prescribed tolerance.

In summary, an optimization framework for flapping kinematics and period modulation is established. By leveraging the discrete adjoint method, efficient gradient evaluation in a high-dimensional design space is achieved, enabling the computation of physically realizable optimal kinematics under power constraints. In the next section, numerical examples are presented to demonstrate the performance of the proposed optimization method under different weighting parameters.

## 4. Results and Discussion

In this chapter, the parameter-identified Morison hydrodynamic model is first systematically compared with high-fidelity CFD simulations to validate the reliability of the low-order model in predicting thrust and input power under typical sinusoidal flapping motions. Based on this validation, the resulting kinematic optimization outcomes obtained via the discrete adjoint method are presented and subsequently back-tested using CFD simulations. By comparing propulsive performance and energy consumption before and after optimization, the consistency between the low-order model predictions and high-fidelity CFD results is analyzed.

### 4.1. Parameter Identification Results

To assess the applicability of the low-order hydrodynamic model based on the Morison equation proposed in Section 2.2, high-fidelity CFD simulations are first employed for parameter identification and comparative analysis of the hydrodynamic forces acting on the flapping foil. All simulations and subsequent kinematic optimizations are conducted using a rectangular flat plate with a chord length of $l_{c}=0.16\;m$, a span length of $l_{s}=0.18\;m$, and a uniform thickness of 0.003m, ensuring that the obtained results possess clear engineering relevance and good comparability.

It should be noted that, for unsteady flapping-foil hydrodynamics, it is generally difficult for the Morison equation, as a low-order model, to accurately reproduce all motion regimes characterized by different combinations of frequency, amplitude, and phase using a single set of model parameters [14,15]. Moreover, numerous experimental and numerical studies based on kinematic optimization and brute-force parameter sweeps have demonstrated that, under sinusoidal heave and pitch kinematic constraints, the Pareto front formed by the mean thrust and the input power typically concentrates around a phase difference close to $90^{\circ }$ [4,28]. This region is widely regarded as a compromise between propulsion efficiency and power utilization. Based on these considerations, the parameter ranges listed in Table 1 are selected for the identification of the Morison hydrodynamic model, with a uniform inflow velocity of $U_{\infty }=0.3\;m/s$. Accordingly, the Reynolds number defined as $Re=U_{\infty }l_{c}/\nu$ remains constant at Re=48,000 for all CFD cases. The corresponding Strouhal number, defined as $St=2fA/U_{\infty }$, varies within the range of St = 0.01∼8.17, where *A* denotes the maximum trailing-edge amplitude (i.e., half peak-to-peak displacement).

During the parameter-identification process, the Morison coefficients are determined using time-resolved hydrodynamic-force data rather than cycle-averaged quantities, such that the reduced-order model can better reproduce the instantaneous force evolution associated with the local velocity and acceleration states. In addition, sinusoidal kinematics with multiple frequencies, amplitudes, and phase differences are employed during calibration to improve the robustness of the identified coefficients over a broader range of unsteady motion conditions and temporal variations. The use of sinusoidal inputs also facilitates systematic traversal of the parameter space while maintaining physically interpretable excitation patterns for the reduced-order-model identification process.

**Figure 3:**
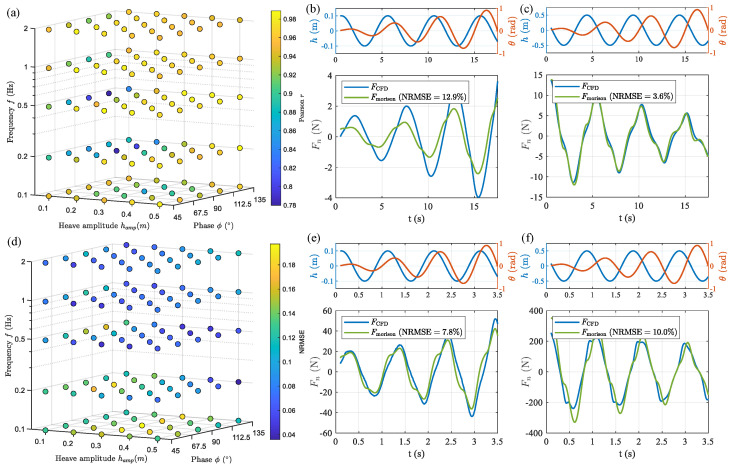
Comparison between the identified Morison model and CFD results. (**a**) Pearson correlation coefficient between $F_{\mathrm{Morison}}$ and $F_{\mathrm{CFD}}$ under different combinations of flapping frequency, heave amplitude, and phase difference. (**d**) Normalized root mean square error (NRMSE) for the same parameter set. (**b**,**c**,**e**,**f**) Time histories of the normal force $F_{n}$ obtained from the Morison model and CFD simulations. (**b**) Case 1 (f=0.2Hz, $h_{\mathrm{amp}}=0.1\;m$, $\phi =45^{\circ }$), (**c**) Case 2 (f=0.2Hz, $h_{\mathrm{amp}}=0.5\;m$, $\phi =135^{\circ }$), (**e**) Case 3 (f=1.0Hz, $h_{\mathrm{amp}}=0.1\;m$, $\phi =45^{\circ }$), (**f**) Case 4 (f=1.0Hz, $h_{\mathrm{amp}}=0.5\;m$, $\phi =135^{\circ }$).

Figure 3a,d present the Pearson correlation coefficient and the normalized root mean square error (NRMSE) between the hydrodynamic forces predicted by the Morison model, $F_{\mathrm{Morison}}$, and those obtained from CFD simulations, $F_{\mathrm{CFD}}$, respectively. These two metrics are employed to evaluate the model performance from the perspectives of temporal trend consistency and force magnitude error.

The Pearson correlation coefficient is defined as

(33) $$r=\frac{\sum_{j=1}^{N}\left(F_{\mathrm{Morison},j}-{\bar{F}}_{\mathrm{Morison}}\right)\left(F_{\mathrm{CFD},j}-{\bar{F}}_{\mathrm{CFD}}\right)}{\sqrt{\sum_{j=1}^{N}{\left(F_{\mathrm{Morison},j}-{\bar{F}}_{\mathrm{Morison}}\right)}^{2}\sum_{j=1}^{N}{\left(F_{\mathrm{CFD},j}-{\bar{F}}_{\mathrm{CFD}}\right)}^{2}}}.$$

The NRMSE is defined as

(34) $$\mathrm{NRMSE}=\frac{\sqrt{\frac{1}{N}\sum_{j=1}^{N}{\left(F_{\mathrm{Morison},j}-F_{\mathrm{CFD},j}\right)}^{2}}}{F_{\mathrm{CFD},\max}-F_{\mathrm{CFD},\min}}.$$

It can be observed that, for all tested parameter combinations, the Pearson correlation coefficient remains above 0.78, indicating that the identified Morison model is capable of capturing the overall temporal evolution and dominant frequency content of the flapping-foil hydrodynamic forces. However, relatively low NRMSE values are only obtained for cases with larger heave amplitudes and higher flapping frequencies, whereas the error increases significantly in low-amplitude and low-frequency regimes. This observation further confirms that the Morison model can only serve as an approximate representation of flapping-foil hydrodynamics within a limited parameter range.

To provide a more intuitive comparison of the fitting performance under different operating conditions, Figure 3b,c,e,f show the time histories of the normal force $F_{n}$ for four representative cases. Case 1 corresponds to f=0.2Hz, $h_{\mathrm{amp}}=0.1\;m$, and $\phi =45^{\circ }$; Case 2 to f=0.2Hz, $h_{\mathrm{amp}}=0.5\;m$, and $\phi =135^{\circ }$; Case 3 to f=1.0Hz, $h_{\mathrm{amp}}=0.1\;m$, and $\phi =45^{\circ }$; and Case 4 to f=1.0Hz, $h_{\mathrm{amp}}=0.5\;m$, and $\phi =135^{\circ }$.

In Case 1, a pronounced discrepancy in force amplitude can be observed between the Morison prediction and the CFD results, and the force signal obtained from the Morison model exhibits a noticeable phase lead. By contrast, in Cases 2–4, the two results show good agreement in terms of dominant amplitude and phase. Although local discrepancies still exist, the Morison model is able to reasonably reproduce the overall temporal evolution and oscillation trends observed in the CFD results, which is further reflected by the relatively lower NRMSE values and high Pearson correlation coefficients obtained in these cases. Nevertheless, the Morison-predicted force histories display several non-smooth turning points. This behavior primarily arises from the fact that the Morison equation represents the hydrodynamic force as a linear combination of an acceleration-dependent term (added-mass force) and a quadratic velocity-dependent term (drag force). The intrinsic phase difference between velocity and acceleration prevents a smooth force transition throughout the flapping cycle. Moreover, the underlying physical assumptions of the Morison model neglect unsteady flow mechanisms such as vortex shedding, vortex–vortex interactions, and flow-history effects. Consequently, the model is not derived from the evolution of the flow field but rather constitutes an equivalent parametric approximation of complex hydrodynamic behavior.

Based on the above analysis, it can be concluded that, within a certain range of kinematic parameters, after appropriate parameter identification, the Morison model is capable of reconstructing the flapping-foil hydrodynamic response in a statistical and trend-consistent sense. This property makes it particularly suitable for rapid hydrodynamic evaluation and relative performance comparison in kinematic optimization studies.

To further quantify its applicability, the NRMSE is employed as an empirical indicator to characterize the agreement level between the Morison-based predictions and CFD results, while the Pearson correlation coefficient is additionally used to evaluate waveform consistency and temporal trend similarity. Since the Morison formulation does not explicitly resolve vortex shedding and wake evolution, the present validation focuses primarily on trend-level consistency rather than strict pointwise agreement of instantaneous force amplitudes. In the present study, cases with NRMSE≲10% are generally regarded as exhibiting reasonably consistent trend agreement between the reduced-order model and CFD results. Under this framework, the Morison model exhibits reasonably consistent trend prediction capability for f=0.2Hz with $h_{\mathrm{amp}}≳0.4\;m$, for f=0.5Hz with $h_{\mathrm{amp}}≳0.2\;m$, and generally for cases with f≲2Hz. It should be noted that these bounds arise from a coupled multi-parameter effect, and therefore do not represent strict independent limits on individual parameters. In terms of the corresponding dimensionless characterization, these cases collectively correspond to a Strouhal number range of St = 0.35∼8.17, representing the regime where the Morison-based model provides qualitatively reliable agreement and trend consistency with CFD results under the present formulation.

Moreover, for non-parameterized kinematic motions, the resulting trajectories may exhibit multiple inflection points in velocity and acceleration profiles, which fall outside the smooth harmonic patterns considered here. As such, the above applicability range cannot be directly used as a strict constraint in non-parameterized optimization, and the optimized results should still be validated using high-fidelity CFD simulations.

The identified model parameters are $C_{D_{n}}=2.49$, $C_{D_{t}}=3.12$, $C_{a_{n}}=1.02$, $C_{a_{t}}=0.87$. which will be employed in the subsequent section for flapping-foil kinematic optimization and propulsion performance assessment.

### 4.2. Adjoint-Based Optimization Results

This section presents the flapping-foil kinematic optimization results obtained using the discrete adjoint method. All optimization results are based on the Morison hydrodynamic model identified in the previous section and are obtained under a prescribed instantaneous input power constraint of $P_{\max}=50\;W$.

**Figure 4:**
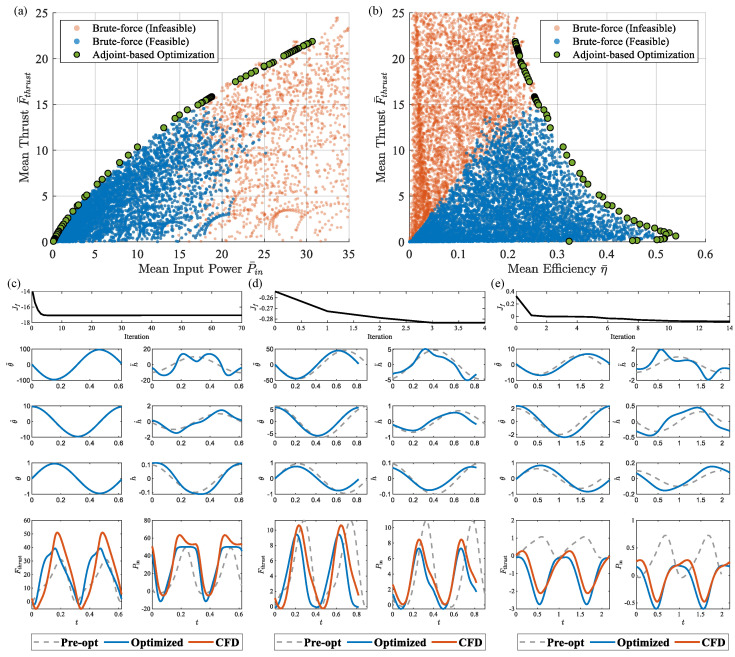
Optimized flapping kinematics obtained using the adjoint-based method under the instantaneous power constraint $P_{\max}=50\;W$, showing the temporal evolution of heave and pitch motions. (**a**) Relationship between cycle-averaged thrust and cycle-averaged input power. (**b**) Relationship between cycle-averaged thrust and cycle-averaged propulsive efficiency. (**c**–**e**) Optimization results for β=0, β=0.5, and β=1.

We first employ the identified Morison model to perform a brute-force parameter sweep over sinusoidal kinematics, evaluating the flapping-foil propulsion performance in terms of the cycle-averaged thrust, cycle-averaged input power, and cycle-averaged propulsive efficiency. The resulting thrust–input power and thrust–efficiency relationships are shown in Figure 4a,b, respectively. The cycle-averaged quantities are defined as

(35) $${\bar{F}}_{\mathrm{thrust}}=\frac{1}{N}\sum_{j=1}^{N}F_{\mathrm{thrust},j},\;{\bar{P}}_{\mathrm{in}}=\frac{1}{N}\sum_{j=1}^{N}P_{\mathrm{in},j},\;\bar{\eta }=\frac{U_{\infty }\;{\bar{F}}_{\mathrm{thrust}}}{{\bar{P}}_{\mathrm{in}}}.$$

In Figure 4a,b, red markers indicate sinusoidal kinematic cases that violate the instantaneous input power constraint, which are primarily concentrated in the high-frequency region of the parameter space. Blue markers correspond to feasible cases that satisfy the power constraint and collectively form a constrained Pareto front within the sinusoidal kinematic space. This constrained Pareto front is used as the initial solution set for the discrete adjoint optimization in order to accelerate convergence. A clear separation can be observed between the red and blue markers, indicating that the instantaneous input power constraint imposes a strict upper bound on the maximum achievable cycle-averaged thrust for sinusoidal motions.

The green markers represent the solutions obtained from the discrete adjoint optimization. It can be seen that their corresponding cycle-averaged thrust and efficiency values lie outside the constrained Pareto front defined by sinusoidal motions. Although some points appear to fall within the Pareto front in the thrust–efficiency plane shown in Figure 4b, they remain outside the front in the thrust–input power plane shown in Figure 4a. This observation demonstrates that, under the same instantaneous input power constraint, non-sinusoidal kinematics obtained via non-parametric discrete adjoint optimization are able to further expand the achievable efficiency–thrust domain. In particular, when the objective function is solely focused on thrust maximization (β=0), the optimized kinematics achieve a maximum cycle-averaged thrust that is 50.29% higher than that of the best sinusoidal motion. This improvement indicates that the optimization process effectively redistributes the timing and amplitude of the flapping motion, enabling a more efficient utilization of the available instantaneous input power and a better matching between energy input and thrust generation.

Figure 4c–e present comparisons of the kinematics and performance over one flapping cycle before and after non-parametric optimization for three representative weighting coefficients, namely β=0, β=0.5, and β=1. In addition, the optimized kinematics are further validated using CFD simulations, and the resulting thrust and input power are compared against the predictions of the Morison model.

It can be observed that, for all values of β, the discrete adjoint optimization exhibits stable convergence behavior, and the instantaneous input power remains within the prescribed constraint throughout the optimization process.

For β=0 (St=2.12), the optimization objective exclusively prioritizes thrust maximization. Under the input power constraint, the algorithm significantly increases the cycle-averaged thrust. Compared with the initial sinusoidal motion, for which the input power reaches the constraint only at isolated instants, the optimized non-sinusoidal kinematics utilize the available input power more extensively over the entire flapping cycle, thereby generating higher thrust. However, CFD validation reveals noticeable discrepancies between the thrust predicted by the Morison model and that obtained from CFD, and the input power predicted by CFD locally exceeds the prescribed threshold. It should be emphasized that the CFD simulations are not used to enforce or evaluate the optimization constraints, but rather serve as an independent high-fidelity validation tool to assess the robustness and physical consistency of the optimized kinematics under full Navier–Stokes dynamics. This behavior is likely caused by the introduction of multiple oscillations in the heave motion, which are favored by the optimization to saturate the input power constraint but induce complex vortex dynamics and additional energy dissipation mechanisms that are not fully captured by the low-order model. In addition, these locally rapid variations in the optimized acceleration profiles may also pose potential challenges for practical actuation, as actuator bandwidth and motor torque limits are not explicitly modeled in the present optimization framework.

For β=0.5 (St=0.94), the optimized solution achieves a substantial reduction in input power at the cost of only a minor decrease in thrust, resulting in an overall improvement of the weighted objective function. This outcome reflects the balancing nature of the multi-objective weighted optimization. In this case, the CFD results are in good agreement with the Morison model predictions in terms of overall trends, although the Morison-based force signals exhibit a slight phase lead.

For β=1 (St=0.20), the objective function no longer includes a thrust-related term and solely aims to minimize the input power. Since no lower bound is imposed on the input power in the present formulation, the optimization naturally evolves toward extracting energy from the incoming flow. As a result, the cycle-averaged thrust remains negative, and the flapping foil operates in a “generator-like” regime. The CFD results show trends that are generally consistent with those predicted by the Morison model. Because both the cycle-averaged thrust and the cycle-averaged input power are negative in this case, these results are not included in Figure 4a,b.

It is worth noting that, although the optimal control inputs obtained under the three weighting coefficients exhibit markedly different temporal profiles, all of them deviate significantly from conventional sinusoidal forms and display strong non-parametric characteristics. This observation indicates that the discrete adjoint method is capable of identifying high-performance kinematic patterns beyond the prescribed sinusoidal assumption.

The corresponding CFD results are primarily used as an independent physical check on the qualitative consistency of the optimized solutions obtained from the reduced-order Morison model. In particular, CFD is employed to assess whether the overall trends of thrust and input power variation predicted by the surrogate model are preserved under higher-fidelity flow physics, rather than to enforce pointwise agreement in force histories. Within this context, the CFD results generally confirm that the optimized non-sinusoidal motions preserve the overall trends of thrust and input power variation predicted by the reduced-order model, despite the presence of local quantitative deviations caused by unsteady vortex dynamics that are not explicitly captured in the Morison formulation.

**Figure 5:**
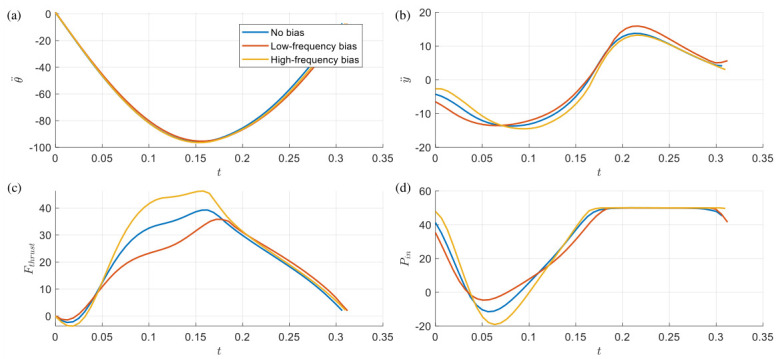
Sensitivity analysis of the optimization results with respect to different Morison-coefficient identification strategies for the (β=0) thrust-maximization case. (**a**) Optimized half-cycle pitching acceleration profiles; (**b**) optimized half-cycle heaving acceleration profiles; (**c**) corresponding thrust histories; and (**d**) corresponding input-power histories.

To further evaluate the sensitivity of the optimization results to the identified Morison coefficients, additional optimization tests are performed using different parameter-identification weighting strategies, as shown in Figure 5. In the baseline identification process (see Figure 3), the Morison coefficients are obtained from sinusoidal flapping datasets spanning five frequencies (f=0.1,0.2,0.5,1, and 2Hz) with uniform weighting. To assess the influence of coefficient variation, two additional identification strategies are introduced: a low-frequency-biased weighting [0.4,0.3,0.15,0.1,0.05] and a high-frequency-biased weighting obtained by reversing the above distribution. The resulting coefficient sets are subsequently applied to the β=0 thrust-maximization optimization case.

The comparison results indicate that variations in the identified Morison coefficients lead to noticeable differences in the detailed optimized kinematic profiles and quantitative thrust predictions, which is expected since the relative contributions of the added-mass and drag-related terms are modified by the identification process. Nevertheless, the overall optimization trends remain consistent across all identification strategies. In particular, all optimized solutions continue to exhibit strongly non-sinusoidal characteristics with localized redistribution of motion and acceleration, indicating that the proposed non-parameterized optimization framework consistently converges toward similar high-thrust motion patterns despite variations in the identified coefficients. Furthermore, although the predicted thrust levels vary, the corresponding input power remains effectively regulated within the prescribed admissible range for all identification strategies, demonstrating the robustness of the penalty-based optimization framework with respect to moderate coefficient variations. Since the optimized flapping period is also treated as a design variable, the resulting control trajectories possess different temporal evolution patterns, and therefore the present analysis focuses primarily on the robustness of the overall propulsion-performance trends and major kinematic characteristics rather than pointwise trajectory differences.

**Figure 6:**
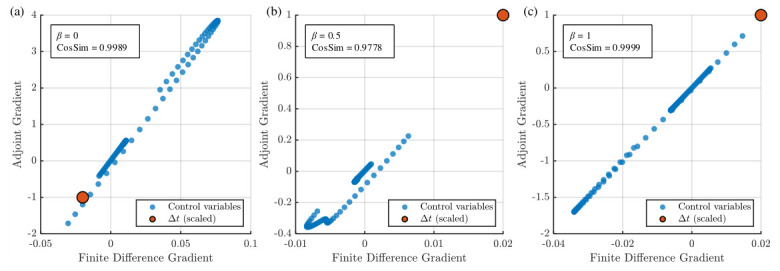
Comparison between adjoint-based and finite-difference (FD) gradients evaluated at the same initial design point under different weighting coefficients: (**a**) β=0; (**b**) β=0.5; (**c**) β=1.

To further verify the correctness of the discrete adjoint implementation, additional gradient-validation tests are performed by comparing the adjoint-based gradients with independently computed finite-difference (FD) gradients under the three weighting conditions β=0, β=0.5, and β=1. Figure 6 presents the corresponding scatter comparisons between the adjoint and FD gradients evaluated at the same initial design point. Since the gradient component associated with the time-step variable Δt exhibits a substantially different magnitude from the remaining control-related gradients, direct visualization would cause the Δt contribution to dominate the scatter distribution. Therefore, for visualization purposes only, the Δt gradients from both methods are normalized using

(36) $$\Delta t_{\mathrm{scale}}=\max\left(|g_{\Delta t}^{adj}\left|,\right|g_{\Delta t}^{FD}|\right),$$

followed by

(37) $$g_{\Delta t}^{adj}\leftarrow g_{\Delta t}^{adj}/\Delta t_{\mathrm{scale}},\;g_{\Delta t}^{FD}\leftarrow g_{\Delta t}^{FD}/\Delta t_{\mathrm{scale}}.$$

Here, $g^{adj}$ and $g^{FD}$ denote the adjoint and finite-difference gradient vectors, respectively. The subscript Δt represents the gradient associated with the time-step variable, while the subscript u denotes the gradients associated with the discrete control variables.

To quantitatively evaluate the consistency between the two gradient-evaluation approaches, the cosine similarity is additionally computed as

(38) $$\mathrm{CosSim}=\frac{{g_{u}^{adj}}^{T}g_{u}^{FD}}{∥g_{u}^{adj}∥\;∥g_{u}^{FD}∥}.$$

In this calculation, the Δt component is excluded in order to avoid the similarity metric being dominated by the large magnitude difference associated with the time-step gradient. The resulting cosine similarities are 0.9989, 0.9778, and 0.9999 for β=0, β=0.5, and β=1, respectively. The scatter distributions exhibit a clear proportional relationship of the form y=kx, indicating strong directional consistency between the adjoint and FD gradients. Although the β=0.5 case shows slightly larger scatter than the other two cases, the overall agreement remains very strong, demonstrating that the discrete adjoint method correctly captures the dominant gradient directions of the optimization problem.

In summary, the gradient-validation results confirm the numerical consistency and correctness of the proposed discrete adjoint implementation. Furthermore, the results demonstrate that the proposed non-parametric discrete adjoint optimization framework converges robustly under instantaneous input power constraints and outperforms traditional sinusoidal-based approaches in terms of the thrust–energy trade-off. This advantage stems from the expanded design space enabled by the non-parametric formulation, which allows flexible redistribution of motion amplitude, phase, and timing over the flapping cycle, leading to localized actuation patterns that are not attainable with conventional parameterized methods. The corresponding CFD validation further indicates that the proposed framework is capable of capturing the dominant trends of thrust and power variation under different optimized kinematic patterns, although some highly oscillatory optimized motions may also introduce more complex vortex dynamics and flow-history effects that are not fully represented by the present Morison-based reduced-order model.

## 5. Conclusions and Future Work

This study has investigated non-parametric flapping-foil kinematics optimization using the discrete adjoint method, built upon a Morison-based low-order hydrodynamic model calibrated and validated against high-fidelity CFD simulations. The results indicate that the optimized flapping motions can substantially enhance propulsion performance while satisfying instantaneous power constraints, producing non-sinusoidal, high-efficiency motion patterns. Compared with conventional sinusoidal motions, the optimized kinematics achieve up to 50.29% increase in mean thrust in thrust-maximization cases, demonstrating that the optimization effectively redistributes the amplitude and timing of heave and pitch to better utilize the available instantaneous power. Optimization with different weighting factors further reveals the thrust–power trade-off and consistently yields improved performance over parameterized sinusoidal motions. Overall, the proposed framework demonstrates the capability of gradient-based discrete-adjoint optimization for high-dimensional time-resolved flapping-motion design. By directly optimizing the full time-discretized kinematic history under explicit physical constraints, the method provides a computationally tractable approach for exploring non-sinusoidal high-efficiency flapping motions beyond conventional parameterized kinematic formulations.

The calibrated Morison model reproduces the hydrodynamic response of the flapping foil in a statistically consistent sense and is particularly suitable for rapid evaluation and relative comparison of different kinematic schemes. Pearson correlation coefficients exceed 0.78 across various combinations of flapping frequency, heave amplitude, and phase difference, indicating that the model captures the dominant periodic features and phase relationships. The error distribution shows that the NRMSE remains lower at high-frequency, large-amplitude conditions and increases at low-frequency, small-amplitude conditions. CFD validation further confirms that the model can reasonably predict the trends in forces and power for the optimized motions, although local deviations may occur under strongly unsteady conditions due to the inability of the reduced-order formulation to explicitly resolve detailed vortex dynamics, wake interactions, and flow-history effects. Therefore, the present Morison-based framework should be regarded as a computationally efficient reduced-order surrogate for gradient-based time-resolved kinematic optimization, rather than a high-fidelity vortex-resolving flow model.

Future work will extend the present framework by incorporating more advanced low-order hydrodynamic models capable of representing vortex dynamics, such as discrete vortex methods (DVMs), together with fully coupled robotic fish dynamics and actuator dynamics within the forward propagation process of the discrete adjoint framework to better capture vortex shedding, unsteady flow effects, and practical motion realizability. To further validate the proposed optimization framework, the optimized flapping kinematics will be implemented on a real robotic fish, allowing experimental assessment of the predicted performance improvements. In addition, exploring energy recovery and optimal power control strategies as well as incorporating actuator-level constraints such as motor torque limits, bandwidth restrictions, and motion realizability requirements may further enhance the efficiency and endurance of bio-inspired underwater propulsion systems.

## Figures and Tables

**Table 1:** Parameter ranges used for the identification of the Morison hydrodynamic model.

$h_{\mathrm{amp}}$ (m)	0.1, 0.2, 0.3, 0.4, 0.5
ϕ (°)	45, 67.5, 90, 112.5, 135
*f* (Hz)	0.1, 0.2, 0.5, 1, 2

## Data Availability

The data generated during the current study are available from the corresponding author on reasonable request.
